# Supplementary material for: Health-Related Gender Knowledge: Scale Development and Validation in Spanish Nursing Students
Source: Nurs Rep. 2025 May 27;15(6):187. doi: 10.3390/nursrep15060187 (PMC12196085; doi:10.3390/nursrep15060187)
Supplement: Supplementary file 1 [file nursrep-15-00187-s001.zip › nursrep-3605048-supplementary.pdf]

Table S1. Proportion of answers and item difficulty.

Question	n (%)	ID_1	ID_2
1) Read the following definition: "refers to the biological characteristics of individuals, in terms of reproductive organs and functions based on physiology, chromosomes and hormones". Which concept are we referring to?		**.902**	**.910**
**a) Sex.**	**533 (90.2)**		
b) Gender.	39 (6.6)		
c) Sex and gender are synonymous.	14 (2.4)		
d) I do not know.	5 (0.8)		
2) What percentage of all psychotropic drugs are prescribed to women?		**.372**	**.590**
a) Less than 30%.	16 (2.7)		
b) About 50%.	137 (23.2)		
**c) More than 80%.**	**220 (36.2)**		
d) I do not know.	218 (35.9)		
3) What would you think if a woman presents with epigastric pain, shortness of breath, sweating, and palpitations?		**.616**	**.834**
a) **Acute myocardial infarction.**	**363 (61.4)**		
b) Menstrual pain.	24 (4.1)		
c) COPD.	48 (8.1)		
d) I do not know.	156 (26.4)		
4) Choose the correct option:		**.651**	**.873**
a) Breast cancer only affects women.	45 (7.6)		
b) Men do not have osteoporosis.	11 (1.9)		
c) **Cerebrovascular diseases are more common in women.**	**385 (65.1)**		
d) I do not know.	150 (25.4)		
5) About the gender perspective in medicine, choose the correct option:		**.741**	**.950**
a) It is exclusively concerned with integrating the perspectives and realities of the female population.	5 (0.8)		
b) **It helps to reconsider why and how gender biases can influence the care process.**	**438 (74.1)**		
c) It should be encouraged in male health professionals, as women and non-binary people do not tend to have gender biases.	18 (3.0)		
d) I do not know.	130 (22.0)		
6) Concerning premenstrual dysphoric syndrome:		**.134**	**.246**

a) It affects the majority of women.	59 (10.0)		
b) It affects more than 40% of women.	183 (31.0)		
c) **It affects less than 10% of women.**	**79 (13.4)**		
d) I do not know.	270 (45.7)		
7) Regarding lipedema, please choose:		**.266**	**.872**
a) Its incidence is less than 1 % in men and women.	14 (2.4)		
b) It has no clinical repercussions. It is an aesthetic problem related to obesity.	9 (1.5)		
c) **The World Health Organisation recognised it as a disease in 2018.**	**157 (26.6)**		
d) I do not know.	411 (69.5)		
8) The most appropriate way to present epidemiological data in health sciences is by		**.653**	**.811**
a) **Disaggregating data by sex to identify differences between men, women, boys and girls.**	**385 (65.2)**		
b) Providing aggregate data for the population, as disaggregating by sex is discriminatory.	42 (7.1)		
c) It is sufficient to disaggregate data by sex only for the reproductive system; women and men are physiologically identical in all other systems.	48 (8.1)		
d) I do not know.	116 (19.6)		
9) What is the leading cause of death for each sex? Choose the correct option:		**.205**	**.261**
a) The leading cause of death in women is cancer; in men, it is cardiovascular disease.	246 (41.6)		
b) **The leading cause of death in women is cardiovascular disease; in men, it is cancer.**	**121 (20.5)**		
c) The leading cause of death in both sexes is cancer.	97 (16.4)		
d) I do not know.	127 (21.5)		
10) About pain, choose the correct option:		**.134**	**.184**
a) Women require less analgesia as they are physiologically prepared to withstand pain.	107 (18.1)		
b) **Women are more sensitive to pain.**	**79 (13.4)**		
c) Sex and gender do not influence pain.	244 (41.3)		
d) I do not know.	161 (27.2)		

Note. ID: Item difficulty. ID_1: Proportion of students who answered each item correctly taking into account the total number of students answering each question. ID_2: Proportion of students who answered each item correctly not taking into account the students who have chosen the “I do not know” option.
